# The Impact of Micro and Macro Level Factors on the Working and Living Conditions of Migrant Care Workers in Italy and Israel—A Scoping Review

**DOI:** 10.3390/ijerph18020420

**Published:** 2021-01-07

**Authors:** Oliver Fisher

**Affiliations:** 1 Centre for Socio-Economic Research on Ageing, IRCCS INRCA—National Institute of Health and Science on Ageing, 60124 Ancona, Italy; o.fisher@inrca.it; 2 Department of Economics and Social Sciences, Università Politecnica delle Marche, 60121 Ancona, Italy

**Keywords:** care work, migration, informal care, home care, Italy, Israel

## Abstract

**Background**: The provision of home-based care for frail older adults in Italy and Israel is predominately provided by live-in migrant care workers (MCWs). However, despite the important role that they play in filling the demand for home care, MCWs often experience labor rights violations. This not only impacts the well-being of MCWs but also leads to lower-quality care being provided to people in need of support. **Method**: This scoping review used Arksey and O’Malley’s methodological framework to map literature. This article aims to analyze the scope, main topics, themes and gaps in the existing academic literature on how micro and macro level indicators impact the working and living conditions of live-in MCWs in Italy and Israel. Scopus, Pubmed, and Web of Science Core Collection were searched for peer-reviewed articles. Search terms were adapted from the Multilevel Framework of Transnational Care Migration (MFTCM). Themes were developed using Braun and Clarke’s method for conducting reflexive thematic analysis. Articles were included if they focused on Italy and/or Israel, included analysis on the working and living conditions of live-in MCWs at the macro and/or micro levels, were written in English, and were published between 2015 and 2020. **Results**: Out of the 1088 articles retrieved, 33 met the inclusion criteria. A total of 18 articles focused on Italy and 14 on Israel, and one focused on both Italy and Israel. The majority of articles in Italy (84 per cent) and Israel (53 per cent) included analysis on care regimes. Only 37 per cent of articles in Italy and 20 per cent in Israel included analysis on gender regimes. At the micro level, 80 per cent of articles in Israel discussed Power/Class Asymmetry, compared to 37 per cent in Italy. In total, six themes were developed. At the macro level, these themes included funding care work, MCWs as a pragmatic approach, care in the home, and valuing care work. At the micro level, the themes included being part of the family, and perceptions on class asymmetries. The findings presented in this review show that MCWs in both Italy and Israel face many of the same challenges in accessing decent work opportunities, despite contrasting employment and migration policies in each country. This can be partially attributed to the undervaluing of care work because of racialized and gendered notions of care. At the macro level, this has contributed to a lack of political will to develop long-term sustainable solutions to create or monitor decent work standards for MCWs. At the micro level, this has led to power imbalances between MCWs and people in need of care and their family members, resulting in MCWs being expected to work hours beyond those contractually allowed, having little to no time off, and experiencing emotional, physical, and sexual abuse. **Conclusion**: This study provides a review of the most recent contributions to the fields of labor migration and health concerning the MCW markets in Italy and Israel. While there have been many studies in each country that detail the labor rights violations experienced by MCWs, this is the first review that develops themes around the underlying causes of these violations. By thematically analyzing the findings of recent studies and current gaps in existing knowledge, this scoping review assists in building the groundwork for the development and implementation of policy, strategies, practice and research to improve the rights and migration experiences of MCWs.

## 1. Introduction

### 1.1. Background

Home-based care—defined as any form of assistance provided to a patient directly in the home [1]—is often preferred to institutional care by people in need of support [2]. At the same time, many states promote home-based care due to the high costs associated with institutional care. Consequently, informal caregivers—defined as people that provide any type of care or help (often unpaid) to older adults (family or otherwise), working-age adults, young people and children with disabilities, and those living with mental health problems [3]—are frequently relied upon to provide support for older adults and to fill long-term care gaps. It is estimated that informal caregivers provide up to 80 per cent of all long-term care in Europe [4]. Informal caregivers provide important contributions to societal welfare. Nevertheless, the support they provide involves substantial time costs and can negatively affect their mental and physical health and well-being [5,6]. Studies have shown that informal caregivers have higher rates of depression, lower levels of well-being, and experience more cardiovascular problems than those not providing informal care [7,8,9]. These negative effects can result from the stress caused by trying to meet the competing demands of their family and paid work, social exclusion, and the physically demanding nature of providing care [10]. These factors are further compounded for informal caregivers that share the same household as the person in need of support, due to a higher intensity of caregiving [11].

The demand for home-based care for older adults is rising across many high-income countries [12]. From the demand side, this has largely been driven by a combination of changes in demographic structures, including increasing life expectancies, and the introduction of policies (e.g., cash for care schemes or in-kind care allowances) that enable families or individuals to hire non-familial workers through the market [2,13]. On the “supply” side, the availability of informal caregivers is expected to decrease in the coming years, due to a generalized drop in intergenerational co-residency, increases in women’s labor market participation rates—which has led to a rise in the number of dual-income families, but not to an increase in men providing informal care—and rising old-age dependency ratios [14]. Projections to 2060 have estimated that the supply of informal caregivers is unlikely to match the demand for care by older adults [15].

Both Italy and Israel face challenges in providing long-term care to older adults due to their ageing populations. In Italy, 23.3 per cent of the population are over the age of 65 and the number of people over the age of 85 is estimated to have increased by 80 per cent between 2005 and 2020 [16]. In Israel, 12.4 per cent of the population are over the age of 65, and the number of people over the age of 85 has increased by 51 per cent between 2005 and 2020 [16]. Consequently, the demand for long-term care for older adults is likely to remain high for both countries in the coming decades.

Families in both Italy and Israel have turned to hiring migrant care workers (MCWs) to fill long-term care gaps in home-based care [17]. In 2019, there were 848,987 care and domestic workers in Italy, of which 596,964 (70.3 per cent) were migrants [18]. Over half of the MCWs in Italy with regular migration status were from Eastern Europe, with the next most common groups being from South America and Northern Africa [18]. Moreover, the number of MCWs in Italy is often underestimated due to the large number employed in the informal economy (The informal economy refers to “all economic activities by workers and economic units that are—in law or in practice—not covered or insufficiently covered by formal arrangements” [19].) [20]. In 2019, there were 57,111 MCWs with regular migration status and 12,145 with irregular migration status providing home-based care in Israel [21]. The majority of MCWs in Israel with regular migration status are from the Philippines, Nepal, India, Sri Lanka, and Eastern Europe [22]. Most MCWs with irregular migration status in Israel come from Eastern Europe, South Asia, Africa and South America [23].

The MCW market in Italy is mainly funded through unregulated cash for care schemes, with workers hired directly by the person in need of support or their family members [24]. The “indennità di accompagnamento” is the only allowance granted at the national level and is universal and not means-tested [25,26]. As of 2020, recipients receive a flat fee of 520.29 euros a month, rising to 930.99 euros a month if the person in need of support is blind [27,28]. There is no obligation to detail how the money from this allowance is spent. Consequently, as a way to cut costs, many MCWs are hired outside of the formal economy, as employers therefore do not have to pay taxes or social security contributions [29]. In-kind services are less common and are planned at the regional level, but administered at the municipal level [25].

The MCW market in Israel has been built on an in-kind care benefits scheme, with people in need of support allocated a set number of hours of care per week depending on their age, income level, and a needs-based assessment that measures whether the older adult’s level of dependency requires them to have assistance or supervision for most hours of the day [30,31]. Eligible people then receive between 5.5 and 30 care hours per week (26 if hiring a MCW). At the lowest level of dependency, people have the option of exchanging their dependency hours for home care services, long-term services without personal care at home, benefits in cash, or a combination of cash benefits and long-term care services. At higher levels of dependency, people can exchange up to four hours of care for cash benefits, which can be increased to between five and ten hours based on a review [32]. People in need of support that would like to hire a MCW have to first apply for a permit and then, once granted, apply to the Ministry of Internal Affairs and a private care agency to exchange their care benefits [31,33]. Individuals that pass the dependency test but fail the income test are still able to hire a MCW but must pay the total salary of the worker. Individuals also do not pay the salary of the worker directly, but instead pay care agencies with their care hours, or with additional income, if the worker is employed for more hours than are eligible based on the assessment [31]. In comparison to Italy, this system has a higher degree of regulation, which has led to a greater proportion of MCWs being employed in the formal economy [34].

One of the main pull factors for migrants to work in the care sectors in Italy and Israel is that wages are often higher than jobs available in the countries of origin of the MCWs [13,35]. Under the right conditions, care work offers an avenue for improving decent work—defined as work that “is productive and delivers a fair income, security in the workplace and social protection for families, better prospects for personal development and social integration, freedom for people to express their concerns, organize and participate in the decisions that affect their lives and equality of opportunity and treatment for all women and men” [36]—opportunities for migrant workers [13]. However, research from Italy has shown that it is common for MCWs to work excessive hours, while some experience emotional, physical and sexual harassment from people in need of support and their family members [37]. Furthermore, MCWs are routinely undervalued and paid insufficient wages due to asymmetrical relationships with people in need of support and their family members [38]. Findings from Israel have been similar. Green and Ayalon [39], in a study of 338 live-in MCWs, found that almost all participants had experienced workers’ rights violations, including not receiving vacation days, not receiving a weekly day off regularly, and not getting paid sick leave. Likewise, MCWs in Israel are often at risk of losing their regular migration status due to restrictive migration and labor laws, which further puts MCWs at risk of exploitation or abuse [31]. Additionally, MCWs often have few trusted or available avenues for reporting labor rights violations [39].

The exploitative working conditions and labor rights violations experienced by MCWs have longstanding and negative impacts on the well-being of workers. In the case of Italy, returned MCWs have been identified by Ukrainian psychiatrists as having a specific depressive disorder called “Italy syndrome”, which is caused by the exploitative living and working conditions experienced by workers in the care sector [40]. In Israel, Ayalon [41], in a study that included 178 Filipino MCWs, found that since their arrival in Israel, 24% reported that they felt their life was not worth living, 7.9% reported that they wished they were dead and 14.6% reported seriously considering taking their own life.

The Multilevel Framework of Transnational Care Migration (MFTCM) [42] is a conceptual model used to analyze and explore transnational domestic and care worker markets and arrangements. This model was developed by Lutz and Palenga-Möllenbeck to provide a cross-national comparative study on care migration from Ukraine to Poland and from Poland to Germany. This model combines approaches from several thematic areas, including migration research, social policy analysis, and gender studies. This model breaks up care migration into the macro, meso, and micro levels. By comparing these levels, this model creates the possibility to investigate to what extent these factors offer opportunities or create obstacles for MCWs.

The macro level of the MFTCM focuses on the intersection of migration, gender, and care regimes. Gender regimes refer to how household and care work organization is seen as an expression of gendered cultural scripts within a society. This determines which tasks and responsibilities are viewed as masculine and feminine. Migration regimes refer to the rules for non-nationals entry into and departure of a country. This ultimately decides which migrants are granted employment, social, political and civil rights and whether they have access to settlement and naturalization. Care regimes include which care responsibilities are distributed between the state, the family, and the market. The meso level examines the organization of care and the role of networks in supporting MCWs and their families. This includes networks made up of MCWs, their families and friends, as well as both formal and informal organizations, for example, private and state recruitment agencies. The micro level focuses on the individual practices, identities, and positions of MCWs. This includes gender-specific characteristics, the migrant’s position in transnational social spaces, and their class and ethnicity. Consequently, the micro level aims to identify the social constructs of masculinity and femininity, ethnicity/class in daily actions, the meanings of private and public workplaces and the gender-specific consequences of migration experiences for women and men migrants [42].

### 1.2. Rationale for Comparing Italy and Israel

Italy and Israel were selected as comparative countries for this review, as they share several common traits concerning the development, demand for, and structure of their MCW markets. First, Italy and Israel are among the countries that have the highest reliance on MCWs to provide home-based care [17,43]. Second, both have familial care regimes, where the majority of care for older adults has traditionally been provided within the family network [31,37]. Third, they share similarities in gender regimes, with women bearing most of the care burden [44,45]. Fourth, both countries are experiencing rapid population ageing, which is likely to result in a high level of demand for home-based care in the coming years [16]. Fifth, the two countries have a relatively large share of older adults that require care and report unmet long-term care needs [46]. Lastly, public expenditure on long-term care support is low in both countries [43].

In contrast, historical differences in cultural, political, and economic development have led to divergences between Italy and Israel at the macro level in the provision of public benefits to support the care market, and their migration and employment laws. At the micro level, these differences can result in deviations in how class, ethnicity and gender dictate the power dynamics between MCWs and people in need of support and their family members. Consequently, by comparing the similarities and differences between the two countries, it is possible to gain a greater understanding of how different micro and macro level factors are influencing the working and living conditions of MCWs in each country and which best practices, if any, can be drawn from these two cases.

### 1.3. Aims and Research Question

This article aims to provide an overview of how micro and macro level indicators detailed in the MFTCM impact (both positively and negatively) the working and living conditions of live-in MCWs. In this regard, this study has the following research question:

*What are the scope, main topics, and gaps in the existing academic literature on how micro and macro level indicators detailed in the MFTCM impact the working and living conditions and opportunities of live-in MCWs in Italy and Israel?*

## 2. Materials and Methods

### Study Design

This article uses Arksey and O’Malley’s methodological framework for conducting scoping reviews [47]. This methodology follows a five-step framework:

1. Identifying the research question;
2. Identifying relevant studies;
3. Study selection;
4. Charting the data;
5. Collating, summarizing and reporting the results.

MCW literature spans a wide range of fields, including migration and immigration studies, sociology, health and ageing, anthropology, politics, and social policy. The scoping review methodology outlined by Arksey and O’Malley [47] has been chosen for this article, as it allows for a rigorous and transparent method for mapping this broad range of research. Likewise, this approach has also been designed to allow for the identification of research gaps in the existing literature. This scoping review also follows the Preferred Reporting Items for Systematic reviews and Meta-Analyses extension for Scoping Reviews (PRISMA-ScR) Checklist (see Appendix A). No review protocol exists for this study.

**Stage 1. Identifying the research question**

The research question used in this scoping review was identified through a preliminary search of existing literature on MCW markets in Italy and Israel. It quickly became apparent that there is a wide range of literature available in both countries on the working and living conditions of MCWs. However, there have not been any review articles which develop themes in the existing literature on which aspects positively or negatively affect the working and living conditions of MCWs in each country.

From a labor rights framework, both Italy and Israel have ratified the 8 core ILO conventions under the Declaration on Fundamental Principles and Rights at Work, while Italy has also ratified the ILO Domestic Workers Convention (No. 189) (C189). Consequently, both countries have an obligation to uphold the labor rights of MCWs. Government officials and practitioners that work on MCW issues in both Italy and Israel need up-to-date information about how government interventions and policies, and the individual actions between MCWs and people in need of support and their family members influence the working and living conditions of MCWs. Identifying these factors will therefore assist in developing strategies and frameworks to improve the working conditions of MCWs.

The poor working conditions in the care sector not only impact the well-being of MCWs but also reduce the quality of care being provided to people in need of support [13,48]. The reliance on MCWs to provide home care in both countries is likely to continue in the future due to demographic trends. Therefore, if Italy and Israel want to ensure high-quality care for their citizens, it is important to improve the working conditions of MCWs.

The MFTCM was identified as an appropriate analytical model to use in this review as it combines themes from a wide range of thematic areas. While this framework analyses transnational migrant care work from the macro, meso, and micro levels, this article only analyses the macro and micro levels to allow for a sharper comparison in results.

**Stage 2. Identifying relevant studies**

Due to the interdisciplinary nature of research on migrant care work, several electronic databases from different fields, including Scopus, Pubmed, and Web of Science Core Collection, were used to search for articles (the most recent search was executed in July 2020). As the MCW field is one that is constantly changing and is also largely dictated by policy and regulatory frameworks, only articles published between 2015 and 2020 were included. This allows for a more recent and up-to-date comparison of the literature.

The keywords used in this review were based on the micro and macro levels of the MFTCM. These included:

Macro level: gender regime, migration regime, care regime, welfare state, care culture, employment regime;

Micro level: gender, class, ethnicity;

Actions/Actors: informal caregiving, informal caregiver, family caregiver, migrant care worker, migrant domestic worker, domestic worker, care worker, migrant.

The full search terms, including the Boolean operators used, can be found in Appendix B.

**Stage 3: Study selection**

Texts were included in this scoping review if they provided qualitative, quantitative or mixed-methods analysis on factors that influence the working or living conditions and experiences of MCWs. Articles were only included if they focused on live-in migrant care work and if they provided analysis at the micro (individual practices and identities) or macro (regimes) levels. Texts had to include analysis specifically on Italy and/or Israel. Articles that included analysis on other countries were also included, provided some of the analysis was on Italy or Israel and that it was possible to separate the data and findings that focused on one or both of the countries. Only texts included in peer-reviewed journals were included. This was decided as it was not always possible to access full versions of certain books and grey literature. Likewise, only texts that were written in English were included, due to language limitations of the researcher who conducted this review.

**Figure 1:**
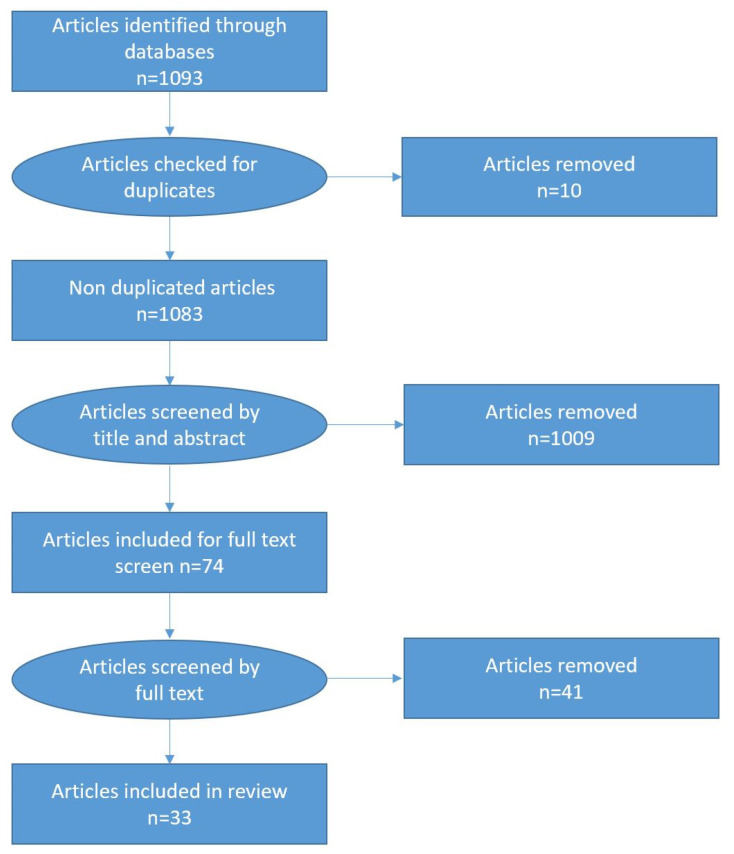
Search process of identified articles.

In total, 1093 texts (see Figure 1) were identified through the electronic databases. As there was overlap in the articles found in the databases, EndNote software was used to remove any duplicate articles. This resulted in ten articles being excluded. Following this, a three-step process was conducted to determine which articles were included in the final analysis. In the first step, articles were screened by both title and abstract to determine whether they met the inclusion criteria. This resulted in 74 articles being selected. In some cases, due to a lack of information available in the title or abstract, it was not possible to determine whether the inclusion criteria were met. Consequently, these articles were included in the full-text screening, alongside other articles that met the inclusion criteria. Following the full-text screen, 41 articles were excluded, resulting in 33 articles being included in this review (see Appendix C for the bibliography of articles included).

**Stage 4. Charting the data**

**Overall characteristics of the articles**

To organize the data included in the studies, data charts were created. The first chart (Table 1 below) focuses on the characteristics of the articles and includes the author(s), year of publication, the field of the journal article, country focus, study populations (e.g., MCWs, people in need of support, expert informants), aims of the study, and methodology used.

Out of the 33 included articles, 18 focused on Italy [29,37,38,44,49,50,51,52,53,54,55,56,57,58,59,60,61,62], 14 focused on Israel [23,39,45,48,63,64,65,66,67,68,69,70,71,72], and one article focused on both Italy and Israel [34].

**Characteristics of the articles focused on Italy**

Five studies were from the field of Migration [37,38,44,56,62], three were from Ageing/Gerontology [29,49,61], two each were from Politics [51,54], and Anthropology [50,61], and one each were from Policy and Society [60], Sociology [59], Health [38], Social Policy [52], Global Studies in Culture and Power [53], Participation and Conflict [57], East and Central Europe [58], and Housing [55].

A total of 16 studies had a qualitative design [37,38,44,50,51,52,53,54,55,56,57,58,59,61,62,67], two were quantitative [29,49], and one study had a mixed-methods qualitative and quantitative design [60].

Two studies were from 2015 [49,65], two were from 2016 [29,51], five were from 2017 [37,38,52,53,54], four were from 2018 [55,56,57,58], and five were from 2019 [44,59,60,61,62].

In terms of participant samples, 11 studies included MCWs [37,38,50,51,53,55,56,58,59,61,62], four included people in need of support [29,49,53,65], and four included key informants [37,44,54,60].

**Characteristics of the articles focused on Israel**

Five studies were in the field of Health [60,61,62,67,68], four Ageing/Gerontology [64,66,68,71], and one each were from Social Policy [72], Social problems [23], Interpersonal Violence [39], Documentation [67], Information, Communication and Society [69], and Feminist theory [45].

Eight studies were qualitative [23,45,63,65,67,69,71,72], five were quantitative [39,48,64,66,70] and two were mixed-method qualitative and quantitative studies [68,71].

Three studies were from 2015 [63,64,65], three were from 2016 [23,39,66], one was from 2018 [48], six were from 2019 [45,67,68,69,70,71], and one study was from 2020 [72].

In terms of participant samples, nine studies included MCWs [45,48,63,65,66,67,68,70,71], seven included people in need of support [48,63,64,65,66,68,71], and five included family members of people in need of support [63,64,65,66,68].

**Characteristics of the article focused on both Italy and Israel**

The one study that focused on both Italy and Israel was from the fields of Ageing/Gerontology and Social Policy. The study sample included key informants, and the study was published in 2020 [34].

**Mapping of micro and macro level factors**

**Figure 2:**
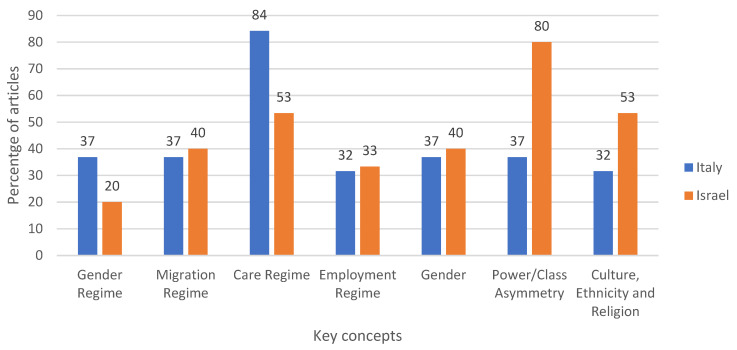
Percentage of articles by country by key concepts.

The second chart (Table 2) focuses on mapping which studies provided findings related to the macro and micro levels of the MFTCM. Figure 2 shows the percentage of articles in Italy and Israel for each key concept of the MFTCM. Employment regime was added at the macro level, which focuses on employment-related laws, policies and regulations. At the micro level, class was broadened to Power/Class Asymmetry and Ethnicity was broadened to Culture, Ethnicity and Religion. A summary of the mapping of keywords is presented below.

**Macro level**

**Key concept 1: Gender regime**

**Articles focused on Italy**

Eight studies focused on Italy (37 per cent) [37,44,52,53,54,55,59,62]. All eight of these studies had a qualitative design. Five of the study samples included MCWs [37,51,53,59,62], two included key informants [44,54], one included people in need of support [53], and one was a content analysis [52].

**Articles focused on Israel**

Three studies focused on Israel (20 per cent) [45,48,65]. Two studies had a qualitative design [45,65] and one was quantitative [48]. Two study samples included MCWs [48,65], one included people in need of support [65] and one included employers of MCWs [45]. (Employers of MCWs can refer to people in need of support and/or their family members. This phrasing is used where the author of the article did not specify how many people in need of support or their family members were included in the article.)

**Key concept 2: Migration regime**

**Articles focused on Italy**

Seven studies focused on Italy (37 per cent) [37,38,52,54,56,61,62]. All seven studies had a qualitative design. Four studies had a sample that included MCWs [37,56,61,62], two included key informants [38,54], and one each included employers of MCWs [61], and content analysis [52].

**Articles focused on Israel**

Six studies focused on Israel (40 per cent) [23,34,39,64,67,69]. Four had a qualitative design [23,34,67,69] and two were quantitative [39,64]. Three study samples included MCWs [39,64,67], one included people in need of support and their family members [64], and one each included employers of MCWs [67], content analysis [69] and key informants [34].

**Key concept 3: Care regime**

**Articles focused on Italy**

Sixteen studies focused on Italy (84 per cent) [29,34,37,38,44,49,50,51,52,53,54,55,58,60,61,62]. A total of 12 of these had a qualitative design [34,37,38,44,50,51,52,53,54,58,61,62], two were quantitative [29,49], and one used mixed methods [60]. Seven studies had a sample that included MCWs [37,50,51,53,58,61,62], five included key informants [34,38,44,54,60], and three included people in need of support [29,49,53], and one each included employers of MCWs [61] and family members [29].

**Articles focused on Israel**

Eight studies focused on Israel (53 per cent) [34,39,45,48,65,68,69,72]. Five of these studies were qualitative [34,45,65,69,72], two were quantitative [39,48] and one was mixed methods [68]. Four samples included MCWs [39,48,65,68], three included people in need of support [65,68,72], two included family members of people in need of support [65,68], and one each included other care workers [48], employers of MCWs [45], and content analysis [69].

**Key concept 4: Employment regime**

**Articles focused on Italy**

Six studies focused on Italy (32 per cent) [37,50,57,58,61,62]. All six of these studies had a qualitative design. For the study samples, five included MCWs [37,50,58,61,62], and one was a content analysis [57].

**Articles focused on Israel**

Six studies focused on Israel (33 per cent) [23,39,48,64,65,69]. Three studies had a qualitative design [23,65,69] and three were quantitative [39,48,64]. For the study samples, four included MCWs [39,48,64,65], and two included people in need of support and their family members [64,65]. One study was a content analysis [69] and one included key informants [23].

**Micro level**

**Key concept 5: Gender**

**Articles focused on Italy**

Seven studies focused on Italy (37 per cent) [37,44,50,51,53,55,59]. All seven studies were qualitative. Six study samples included MCWs [37,50,51,53,55,59], two included key informants, and one each included people in need of support [53], adult children of people in need of support [59], and key informants [44].

**Articles focused on Israel**

Six studies focused on Israel (40 per cent) [23,39,45,48,65,68]. Three had a qualitative design [23,45,65], two were quantitative [39,48], and one used mixed methods [68]. Four study samples included MCWs [39,48,65,68], two included people in need of support and their family members [62,66].

**Key concept 6: Power/Class Asymmetry**

**Articles focused on Italy**

Seven studies focused on Italy (37 per cent) [37,44,50,51,55,59,61]. All seven studies had a qualitative design. Six study samples included MCWs [37,50,51,55,59,61], one included key informants [44] and one included employers of MCWs [61].

**Articles focused on Israel**

A total of 12 studies focused on Israel (80 per cent) [23,45,48,63,64,65,66,67,68,69,70,71]. Six of these studies had a qualitative design [23,45,63,65,67,71], four were quantitative [48,64,66,70], and two were mixed methods [68,71]. Nine study samples included MCWs [48,63,64,65,66,67,68,70,71], and six included people in need of support [63,64,65,66,68,71], and five included family members of people in need of support [63,64,65,66,68].

**Key concept 7: Culture, Ethnicity and Religion**

**Articles focused on Italy**

Six studies focused on Italy (32 per cent) [37,38,44,50,55,56]. All six studies had a qualitative design. Four study samples included MCWs [37,50,55,56], and two included key informants [38,44].

**Articles focused on Israel**

Eight studies focused on Israel (53 per cent) [39,45,48,64,65,67,68,71]. Three had a qualitative design [45,65,67], three were quantitative [39,48,64] and two were mixed methods [68,71]. Seven study samples included MCWs [39,48,64,65,67,68,71], four included people in need of support [64,65,68,71], and three included family members of people in need of support [64,65,68]

**Stage 5. Collating, summarizing and reporting the results**

To develop themes from these articles, this review used Braun and Clarke’s methods for conducting reflexive thematic analysis [73,74]. One limitation of using secondary data for thematic analysis is the absolute dependence on what is in the written record, without the possibility of asking supplementary questions [75]. To reduce the risk of dissociation from the original texts, repeated close readings of each text were conducted.

Findings from each study were initially deductively coded under the micro and macro level indicators detailed in the MFTCM. These headings were represented as parent nodes. This coding process was conducted separately for articles from each country. Since the MFTCM is focused on the interaction between regimes at the macro level and the interaction between individuals at the micro level, it was important to reflect on how codes fit within these interactions, rather than strictly coding under the key phrases from the mapping of articles. Consequently, under the broad subheadings used in the MFTCM, themes were then identified inductively and labelled as child nodes. These themes were identified as being broader patterns within the findings that shared a core concept [73,74]. Once the initial themes were created, the findings from each study were cross-checked with each theme to ensure that the themes were representative of the findings.

## 3. Results

### Themes

In total, six themes were developed. At the macro level, these themes included *funding care work*, *MCWs as a pragmatic approach, care in the home*, and *valuing care work*. At the micro level, the themes included *being part of the family*, and *perceptions on class asymmetries*. Each theme first starts with a summary of the overall theme before analyzing how the findings from the reviewed articles fit into these themes.

**Macro Level**

**Theme 1: Funding care work**

This theme focuses on which mechanisms the government uses to meet the demand for home care. At the care regime level, this includes how home care services are funded, for example through cash allowances or in-kind services. This also covers who can receive these allowances and under which conditions, including any needs-based assessments or financial barriers, as well as any restrictions on how these allowances or services are used. In this regard, low levels of financial support for people in need of support, either in the form of in-kind services or cash allowances can result in MCWs being underpaid, if people in need of support are not able to afford decent wages for MCWs. Furthermore, attaching needs-based restrictions on access to care and services can assist in filtering home care funding to those most in need of support. However, if only those with high levels of dependency receive support, then MCWs are likely to experience a higher level of care burden.

**The Italian context of funding care work**

From the Italian government’s perspective, building a home care market based on the hiring of MCWs is a way to cheaply meet the demand for support in an ageing society [52]. Through this model, the Italian government can shift the cost of home care to people in need of support and their family members, while only providing relatively low levels of finance through cash for care schemes [29,34,52]. This low level of funding often negatively impacts the wages of MCWs, as cash for care allowances do not cover the total costs of hiring a MCW. Therefore, while hiring a MCW may be affordable for some, it is not affordable for all [29]. Consequently, MCWs are often hired outside of the formal economy, as this is cheaper for people in need of support as they are not responsible for paying maternity leave, sick leave and holiday pay [38,50].

The most commonly used cash for care scheme in Italy, the indennità di accompagnamento, does not have any restrictions on how it is used. This reinforces the likelihood of families hiring MCWs outside of the formal sector, as there is no obligation to formally hire workers [29,34,52]. However, in 2012, the National Institute for Social Security launched the Home Care Premium (HCP) scheme to reduce some of the shortcoming of the long-term care system in Italy [52]. The HCP which consists of two measures, the first a national cash for care benefit that covers the cost of employing a live-in care worker with a regular employment contract, and second the provision of in-kind services by local municipalities. The HCP was found to assist in increasing the formality of the MCW market. However, as the HCP was only available to public sector employees or their family members, there were some limitations on its impact [52].

**The Israeli context of funding care work**

To meet the demand for MCWs, Israel has outsourced the management of MCWs to care agencies. Under this system, people in need of support are allocated a certain number of care hours based on a needs, financial and age assessment. People in need of support are then able to exchange their care hours to hire a live-in care worker. This model has resulted in a greater degree of MCWs with regular migration status as people in need of support are required to go through a care agency to hire care workers [23,34,48].

The Israeli care system can increase the likelihood of work-related abuses for MCWs, as only older adults with higher levels of impairment qualify for care hours. This is especially relevant for MCWs who provide care for older adults that have cognitive impairments, as they are more likely to be aggressive than other older adults [48]. Moreover, under this system, only part of the costs of hiring MCWs are covered, which may lead to some people in need of support not being able to hire a MCW or for workers to be expected to provide 24 h care, without increased wages [23].

**Theme 2: MCWs as a pragmatic approach**

At the migration regime level, the state must decide how to structure its migration rules and procedures to facilitate the arrival of migrants into the home care sector. This can include using a formalized care system, with regular migration channels available, or using an ad-hoc regime, which favors choosing to ignore the presence of migrants with irregular migration status. The decision on which path a country takes is often determined by a practical compromise between needing to meet the demand for care and the level of acceptance of migrants within society, which is also based on the gendered and familial norms and expectations around care and whether MCWs are viewed as contributing to the breakdown of these traditional norms. These assumptions then also feed into the employment rules and regulations for MCWs.

**The Italian context of MCWs as a pragmatic approach**

Italy has seen an increase in anti-migration sentiment and xenophobia in recent decades, which can partially be attributed to the rise of the Lega Nord party, a right-wing party that has been part of successive right-wing coalitions since the 1990s [38,54]. The Lega Nord has campaigned on anti-immigration discourse, while also championing women as the ‘mothers of the nation’. Moreover, the party helps to reinforce the notion that women should be responsible for social reproductive work within the home and the family [54].

For some people in need of support, hiring a MCW is a compromise to keep care within the home, even if they would prefer that their children take care of them [38,53]. Hiring a MCW is therefore often viewed as a temporary solution or a less shameful option than living in a nursing home [38,61]. Additionally, MCWs are also sometimes viewed in a positive light, as they are filling gaps in long-term care. However, this acceptance is often determined by the ethnicity of the MCW, with some employers only wanting to hire MCWs from certain countries [37,44,52].

To increase the ease of migrant workers to enter the care sector and despite the anti-immigrant sentiment within Italy, the Italian government has on several occasions made it easier for MCWs to live and work in Italy. Consequently, while workers in other sectors have often faced restrictive migration rules, the Italian government has expressed more leniency towards MCWs [37,38,52,54,57,62]. The Italian government has run several regularization processes, where migrant workers with irregular migration status (e.g., those that entered on tourist visas and overstayed) were able to gain regular migration status through government-led processes [38,54,62].

While regularization processes have assisted MCWs to enter the formal economy, this has been done on an ad-hoc basis, which can increase stress for MCWs as they are unsure when they may gain regular migration status [38,50,54]. This has resulted in MCWs not covered under EU freedom of movement to first enter Italy irregularly and then gain regular migration status at a later date, potentially increasing their risk of exploitation [38,50,54]. Likewise, the cost of the regularization process was too high for some families, leading to some workers not being able to go through the process [44,47,48].

**The Israeli context of MCWs as a pragmatic approach**

The gendered expectation of familialism in Israel has driven the demand for women MCWs in the home care sector. With more Israeli women being employed in the workforce, families have turned to MCWs to fill the care demands of people in need of support [45]. This can also be viewed as a reflection on Israel’s transition into a market-based society. Therefore, while in the past, bringing a non-Jewish care worker would seem ‘unnatural’, it has become more of a common phenomenon as people now have less time to be able to take care of family members [45].

While MCWs are relied on to fill the demand for home-based care in Israel, this has not resulted in MCWs gaining long-term residency rights. The Israeli migration regime has been built on viewing migrant workers as temporary guests [23,34,39,63]. Israeli law has no comprehensive sets of immigration laws or policies that incorporate MCWs into Israeli society. When entering the care section, MCWs are given an Israeli residency permit for 5 years, after which they have to leave the country unless the family they work for asks the immigration office to extend their permit [34]. Consequently, for those that are unable to renew their permit, they must choose between staying in the country with irregular migration status or returning to their home country. Those that choose to stay often face difficulties in finding housing and opening bank accounts, which limits their ability to find new employment [67]. This perception has also shaped rules around family policy, with MCWs not having the right to a family life in Israel, with the expectation that MCWs should arrive and leave alone [64]. Moreover, MCWs are only allowed to be employed on a live-in basis, which further restricts their ability to form a life outside of the workplace [19].

**Theme 3: Care in the home**

Familial care regimes often result in a preference for care being provided in the home, rather than at formal care institutions. This can be viewed as a reflection of the tradition of care being provided by informal caregivers in the home. However, providing care at home results in several complications for MCWs, due to the isolating nature of home-based care. This theme also explores how the home care setting influences the expectations of the types and frequency of care being provided by MCWs. For live-in MCWs, there is often an expectation that care should be provided around the clock. This has serious complications for MCWs, as it limits their ability to take much-needed rest or time off from work. Because MCWs must spend most of their time in the home of the person in need of support, there is often a lack of oversight by labor rights inspectors, which reduces the opportunities for MCWs to report labor rights violations.

**The Italian context of care in the home**

The Italian care regime can be viewed as being built on familial notions of care, where family members have traditionally taken on the role of primary caregivers. Because of the family nature of care, the majority of care has taken place in the home environment and many people in need of support prefer to receive care at home, rather than in an institutional setting [38,50,61].

Providing care in the home can result in labor rights issues for MCWs. Firstly, there is an expectation from both people in need of support and their family members that care should be provided round the clock and that workers are available at all times to provide care. This often-unspoken rule of 24/7 care can lead to MCWs having isolating experiences, with little opportunities to leave the home environment or take time off from their job [44,49,51,53,56,58,60]. This is in contrast to live-out care workers who have more freedom to take time off [50]. Furthermore, the home setting also results in MCWs being free from the oversight of labor rights officials, which makes it difficult for them to report labor rights violations when they take place [50].

**The Israeli context of care in the home**

Proving home-based care presents several barriers to decent work for MCWs in Israel. Providing care can be a socially isolating experience for MCWs, as they often have few opportunities to be able to leave their workplace or to take time off of work, as similar to Italy, there is an expectation for MCWs to provide round the clock care [39,48,64,70]. This can make it difficult for MCWs to be able to form friendships or relationships outside of their work, which can lead to less perceived control among MCWs [64,70].

Ayalon and Green [48] found that live-in MCWs experienced similar levels of work-related abuses as live-out Israeli care workers. While it may have been expected that MCWs would experience increased rights abuses compared to Israeli care workers, due to their lower level of knowledge of the local language, age, and financial burden, the fact that they experience similar levels of work-related abuse suggests that the home-based setting of care work is one of the main factors contributing to the likelihood that MCWs experience labor rights violations.

The home care setting also makes it difficult for workers to report labor rights violations. Social care workers are supposed to monitor MCWs regularly through visits. However, in practice, this may only occur every four months. Moreover, these inspections mainly focus on the quality of care being provided to people in need of support and their well-being, rather than on the well-being of MCWs [39].

**Theme 4: Valuing care work**

This theme explores how values associated with care work influence the type of training and support available to MCWs, as well as how these values shape the adherence towards labor rights for MCWs. Gender stereotypes are common in the home care sector, with many people in need of support and their family members viewing women as ‘natural carers’. This can cause care work to be undervalued and treated as low-skilled work. Negative perceptions in society towards migrants also contributes to this issue, resulting in MCWs having few opportunities to receive skills training or recognition and this can also lead to a lack of understanding of the rights of MCWs by people in need of support and their family members. Additionally, strong familial care regimes can result in the preference to favor the rights of families over paid care workers.

**The Italian context of valuing care work**

The familial care regime present in Italy has resulted in the state preferring to protect the interests of families over MCWs [37,44,54]. Consequently, there has been little political interest in improving the rights of MCWs or tackling exploitation in the sector as this may lead to detrimental consequences for Italian families [37,52,57]. Directive 2009/52/EC on penalties for employers exploiting irregular third-country nationals was developed to address exploitation in the MCW market. However, under this directive, the Italian government chose to add the requirement that only those that hire more than three MCWs are subject to the directive. This was aimed at avoiding punishment for families that hire MCWs outside of the formal market [37].

The Italian government has also ratified ILO C189, which provides minimum standards and rights for domestic workers in Italy [57]. Nevertheless, since its ratification, there have been few attempts to introduce any new legislation to improve the rights of MCWs [57]. Italy also has a National Collective Agreement for Domestic Workers. However, despite MCWs being able to sign permanent contracts under this contract, employers still have the right to terminate contracts [57].

Care work in Italy is often undervalued due to familial and gendered assumptions of care, with the state and families often seeing women as ‘natural carers’ [44]. Families therefore do not place a high value on the skills needed to perform care work, instead preferring to hire workers based on their gender or nationality, rather than work experience [37,44,50,51,53,55]. Subsequently, it is easier for women to be employed as care workers, but harder for men, except in some cases where family members prefer to hire men MCWs to take care of their fathers [44,53].

**The Israeli context of valuing care work**

Ayalon and Green [64] found that employers of MCWs frequently have lack of knowledge about the wages that MCWs were entitled to, which was associated with care work being perceived as a low-skilled and unrewarding occupation. Mazuz [65] supports this notion and argues that the devaluing of care work is often due to the stereotype that women are ‘natural carers’. This undervaluing of care work has led to workers not receiving adequate training for their jobs, which is further reinforced by the understanding that because MCWs are viewed as temporary guests, they are only entitled to elementary rights [63,65]. Moreover, it was reported that people in need of support and their family members often hire MCWs based on racialized and gendered assumptions of MCWs, rather than based on their work experience or skills [45,65].

**Micro Level**

**Theme 5: Being part of the family**

This theme explores how MCWs and people in need of support and their family members navigate their relationship with each other. Due to the personal and close nature of care work, MCWs frequently form close bonds with the person that they provide care for. Consequently, people in need of support often refer to MCWs as being one of the family. These relationships are often ones of fictive kinship, with people in need of support and their family members using the notion of being one of the family as a way to gain control of this relationship and to get workers to perform tasks outside of their usual job. At the same time, MCWs may also choose to lean into this closeness, as a way to gain benefits from the person they provide care for. While other workers prefer to keep their work relationships strictly professional.

The ability of MCWs to negotiate these relationships is often built on the intersection between gender, age, ethnicity and class. How workers navigate these relationships has severe consequences for their well-being, due to how dependent they are on the person they care for to provide them with food and shelter. Building a solid relationship with the person in need of support has the potential to assist MCWs with maintaining their regular migration status and therefore their right to stay in the country, their future job opportunities and the amount of spare time and freedom they have.

**The Italian context of being part of the family**

MCWs and people in need of support often form close bonds with each other due to the amount of time they spend together [55]. However, the asymmetric power relations between the two actors, which are built along the lines of gender, age and ethnicity, often results in this bond disproportionately favoring the person in need of support [37,50,51,55]. It is common for family members and people in need of support to refer to MCWs as being part of the family [37,55]. These relationships are often more in line with fictive kinships, than MCWs actually being considered part of the family [37,55]. Some employers use the idea of the MCW as being one of the family as a way to justify labor rights violations or to try and make MCWs feel that they are being treated as equals, even if this is not the case [37,51].

How MCWs navigate relationships with the person in need of support and their family members has a strong influence on their well-being and rights at work [50,55]. MCWs are reliant on people in need of support for maintaining or forming their regular migration status, the types and intensity of their work, and for future job opportunities [38,50,56,61]. Being close to the person in need of support can in some instances grant MCWs greater flexibility in their job, allowing them to take additional part-time jobs, or to have more say in how they manage their lives outside of the workplace [50].

Becoming too close to the person in need of support can in some instances lead to the person in need of support becoming too attached to the MCW, which limits the amount of free space and time that MCWs have [55]. This can result in some MCWs not wanting to take time off from work, as they worry about the health of the person in need of support when they are not there [61].

MCWs, therefore, have to decide whether they want to try and form professional relationships or whether they want to lean into the narrative that they are part of the family [50,51]. However, this choice is not possible for all MCWs, due to some people in need of support not trusting MCWs because they are migrants or because they would prefer that their children take care of them instead [38,55].

**The Israeli context of being part of the family**

MCWs often show commitment and dedication to the person that they provide care for [45,69,71]. This can result in MCWs being referred to as one of the family by people in need of support and their family members [45,63,68,71]. Being treated as one of the family has both positive and negative consequences for MCWs. On the positive side, it may allow workers to leverage strategic power and avoid inhuman treatment from people in need of support, while also being able to take more time off and build social networks [45]. In contrast, the idea of kinship can also be used by people in need of support and their family members as a way to exert control over MCWs and to get them to do tasks outside of their work responsibilities [45]. Telling MCWs that they are part of the family is a way of maintaining power asymmetries between MCWs and people in need of support. These relationships are often built on fear, racism, class differences, and language barriers, with MCWs often taking a passive role in the caregiver-patient dyad [45,48,63,66,67]. MCWs who have a close relationship with the person in need of support may choose to avoid taking time off because they know that there is no one to replace them to provide care [48]. Consequently, some workers may wave certain rights in favor of caring for the person in need of support [48].

Assumptions on gendered norms of caring also dictate how MCWs are treated by people in need of support. For women MCWs, the failure to perform according to the person in need of support’s desired familial intimacy can result in their dissatisfaction [45]. Women MCWs are also more likely than men to be viewed as one of the family because women MCWs are often viewed as being ‘warm and intimate’ [45]. This can result in women MCWs having difficulties in setting professional boundaries with people in need of support. In contrast, men MCWs often have increased boundaries with people in need of support, as they are less likely to be seen as being one of the family, due to men being viewed as ’less trustworthy’ and ‘not as caring’ [45].

Cultural perceptions around care were also used to justify the structure of the relationship between MCWs and the family members of people in need of support, with some employers viewing themselves as ‘natural managers’ and seeing MCWs as ‘natural caregivers’ [45,48,67]. Age differences can also act as a barrier for MCWs to connect with people in need of support, as generational gaps can lead to differences in preferences for conducting activities [68].

**Theme 6: Perceptions on class asymmetries**

This theme explores how the decision and assumptions around the reasons why MCWs choose to migrate into the care sector are influenced by economic disparities. For many, migration presents an opportunity to send money to family members back home and improve their livelihoods. Assumptions around the rationale for migration are often grounded upon class asymmetry, with people in need of support and their family members using economic disparities as a justification for providing poor working conditions or pay for MCWs. Furthermore, rigid employment laws that do not take into account the short-term goals and the uncertainty around migration, can result in MCWs having to choose between increased labor rights protections or increased income.

**The Italian context of perceptions on class asymmetries**

Increased wages were a common motivation for migrants to work in the care sector, presenting workers with the opportunity to send money to family members back home and to create a better life for their family and children [51,59,61]. Over half of the respondents in a study by Solari [59] stated that escaping poverty was one of their main rationales for migrating. For others, migrating was a way to gain more freedom and autonomy that was not present in their country of origin [62].

Economic motivations can sometimes lead to MCWs losing some of their employment rights. Current pension requirements in Italy require workers to contribute for 10 years before they are eligible to receive pension benefits [58]. Because MCWs are often unsure whether they will stay in Italy long enough to reach eligibility and therefore may choose to work in the informal economy to increase their wages in the short term [58]. This decision can lead to workers losing some rights, including not having an employment contract [61].

Economic disparities between MCWs and people in need of support were used by families to justify the poor treatment of MCWs, with some employers expecting MCWs to be grateful for the opportunity to earn higher wages, even if the workers were being paid low wages [37]. Therefore, although the wages of MCWs were low in comparison to what Italian care workers would earn, employers believed that this was justified as this was more money than workers would earn back in their country of origin [37].

**The Israeli context of perceptions on class asymmetries**

Increased wages were a motivating factor for MCWs to move to Israel [45,67]. Likewise, it was also common for people in need of support and their family members to use this pull factor as a justification for providing poor working conditions or low salaries for MCWs [45]. This can be viewed as a reflection of the perception of class differences between Israeli citizens and MCWs. Some employers believed that they were helping MCWs by hiring them, even if the wages provided to MCWs were low in comparison to Israeli workers [45].

This perception of class disparity was also a source of discontent for some MCWs, as there were many other reasons as to why MCWs decided to move to Israel, including for religious purposes [45]. Thus, some MCWs felt that their existence and identity in Israel was based on assumptions about their financial situation, rather than on their personality or motivations in life [45].

## 4. Discussion

### 4.1. Summary of This Review

In this study, to better understand the scope and gaps in the academic literature on how micro and macro level factors impact the working and living conditions of MCWs, reviewed articles were mapped against key concepts derived from the conceptual model offered by the MFTCM. This mapping assists in identifying topics and areas where future research is needed. Furthermore, four themes at the macro level and two themes at the micro level were developed based on the findings from the reviewed articles.

At the macro level, *funding care work* focuses on the instruments used by governments to meet the demand for home care. This covers who can receive these instruments and under which conditions, as well as whether there are any restrictions on how they are used. *MCWs as a pragmatic approach* discusses the migration rules and procedures used by the government to facilitate the arrival of and long-term residency rights of migrants employed in the home care sector. The theme analyses how these rules are developed based on a compromise between meeting the demand for home care alongside the gendered and familial norms and expectations around care and whether MCWs are seen as contributing to the breakdown of these norms. *Care in the home* explores how the preference in familial care regimes for care to be provided in the home influences the types and frequency of care provided by MCWs. Moreover, it examines how the home cares setting can result in a lack of oversight on the employment conditions of MCWs. *Valuing care work* focuses on how gendered and familial expectations around care alongside perceptions of migrants in society influences if and how the work performed by MCWs is valued.

At the micro level, *being part of the family* discusses how the intersection of age, gender, ethnicity and class determines the types of relationships formed between MCWs, people in need of support and their family members. Additionally, it analyses how and whether MCWs can choose to navigate these relationships. *Perceptions on class asymmetries* analyses how perceptions around class asymmetries between MCWs and people in need of support are used as a justification by people in need of support to justify the poor working conditions of MCWs.

The findings in this review can assist policymakers, labor rights, care, and migration practitioners, researchers, and local authorities in identifying under which areas interventions are needed to improve the working and living conditions of MCWs.

### 4.2. Links to the Existing Literature

As detailed in the theme *funding care work,* the use of unregulated cash for care schemes has contributed to increased informality in the Italian MCW market. In contrast, the more regulated system of using care hours in Israel has resulted in a higher degree of formality in comparison to Italy. These findings are in line with previous research which has shown that the use of unregulated cash for care allowances in Austria, Germany, and Spain, has led to increased informality in their respective home care sectors [2,76]. In comparison, restricting the use of care allowances to buy care services in France and the Netherlands has helped to reduce the number of MCWs employed outside of the formal economy [13,76,77,78].

The findings presented in the theme *MCWs as a pragmatic approach* show that the formalization of the care markets itself does not lead to increased rights if the rights granted under employment and migration regimes are not enforced [79,80]. Taking, for instance, the choice of the Italian government to run regularization processes for MCWs with irregular migration status, while these processes are an effective way to increase the formality of the sector, they do not necessarily lead to increased rights in practice [81]. Similar findings can be found in Thailand, where past research has demonstrated that regularization processes by the Thai government have not been effective in increasing worker’s rights, as the government has not developed a long-term migration policy that addresses the challenges of migrant workers [82,83].

Parallels can be drawn between the migration rules and procedures that govern the MCW markets in Israel and Japan. Both countries view MCWs as temporary guests, which has resulted in the creation of migration policies that offer few avenues for MCWs to stay long-term or to have a right to a family life [84,85,86,87]. Akin to the situation in Israel, the MCW market in Japan has been developed to create a cheap source of care labor, while at the same time minimizing the direct obligation of the state towards the welfare of MCWs [88].

Comparable to the findings displayed in the theme *care in the home*, there is extensive literature that has highlighted that live-in care and domestic workers often experience social isolation and are rarely covered by labor rights inspections [89,90,91,92]. Likewise, it is common in this sector for employers to expect workers to be available 24/7 and to work unpaid overtime hours [93,94,95,96].

The findings presented in this review demonstrate that MCWs in both Italy and Israel face many of the same challenges in accessing decent work opportunities in the care sector, despite the fact that the rules and regulations that govern MCW markets in each country have gone down different paths. As expressed in the theme *valuing care work,* many of the challenges that workers face in Italy and Israel are related to the undervaluing of care work because of racialized and gendered notions of care. The undervaluing of reproductive labor is not unique to Italy or Israel, and many studies in the care and domestic work sectors have evidenced that this undervaluing leads to less recognition and rights for care and domestic workers [97,98,99,100].

This review has also shown in the theme *being part of the family* that the relationship between MCWs and people in need of support and their family members is one of the most important factors that determines whether MCWs have positive or negative employment experiences. Previous studies on the care and domestic sectors in Cyprus, Spain, Malaysia, Thailand and Taiwan have found that fictive kinship relationships are common and that this form of relationship is frequently used by employers to manipulate workers to accept poor working conditions or to perform tasks outside of their usual role [89,101,102]. Moreover, extensive research has detailed that the undervaluing and familial nature of care work may easily lead to expectations that care workers should work excessive hours, beyond those contractually allowed [13,80,103,104].

Similar to the findings presented in the theme *perceptions on class asymmetries*, previous research has shown that the migrant care and domestic sectors are often marked by a high degree of social and economic inequality [105,106,107,108]. Employers frequently discriminate against and justify paying migrant care and domestic workers low wages based on their gender, ethnicity, social class, religion and nationality [89,107,109,110].

### 4.3. Implications for Policy and Practice

This review has identified several key challenges that MCWs face in accessing decent work in the care sector. Nonetheless, it is important to note that care work in itself does not increase the vulnerability of workers, rather it is often a lack of enforcement of labor rights that puts MCWs at risk of labor rights violations. Consequently, there are several steps that the Italian and Israeli governments can take to improve the rights of MCWs.

Concerning the theme *funding care work*, increased funding is needed for people in need of support in Italy to pay for long-term care services. Increasing the amount of funds provided through cash for care schemes would assist people in need of support and their family members to pay MCWs fair wages. Furthermore, this would also help families to meet the requirements set out in Italy’s National Collective Agreement for Domestic Workers [111]. Nevertheless, a simple increase in funding may not lead to an increase in workers being hired through formal channels, unless there are conditions attached to cash allowances. The HCP scheme has shown that requiring people in need of support to hire workers through formal channels is effective, and these conditions could be expanded to the indennità di accompagnamento [29,60].

Linking to the theme *MCWs as a pragmatic approach*, it is important that a more sustainable and long-term route for non-EU MCWs to enter the sector in Italy is created. The current process of relying on ad-hoc regularization processes may assist MCWs in the short term but it also contributes to the exploitation of workers, as it often forces workers to enter the country through irregular channels [38,50,54]. Moreover, these regularization processes require the commitment of people in need of support, which may lead to some MCWs not being able to go through the process of regularization [44,47,48]. The Italian government should therefore develop a channel into the care sector for migrant workers that adheres to the ILO’s General principles and operational guidelines for fair recruitment and definition of recruitment fees and related costs and IOM’s Guidelines for labor recruiters on ethical recruitment, decent work and access to remedy for migrant domestic workers.

MCWs in Israel should be afforded the right to a family life, in line with the International Convention on the Protection of the Rights of All Migrant Workers and Members of Their Families (ICRMW). The current practices of requiring MCWs to leave family members behind in order to migrate into the care sector, or that require MCWs to leave the country if pregnant, or risk losing their regular migration status leads to increased tension and emotional distress for MCWs [23,112].

Allowing MCWs to be employed as live-out care workers in Israel may also assist in improving the living and working conditions of MCWs [113]. The current practice of restricting MCWs to be employed on a live-in basis violates the CEDAW General Recommendation No. 26 as well as ILO C189. Moreover, the Israeli government should also put a plan in place to ratify ILO C189, as this will help to bring labor rights standards in Israel in line with international best practices on domestic worker rights.

Regarding the theme *care in the home*, both the Italian and Israeli governments need to prioritize the enforcement of labor rights for MCWs. While the home setting of care work makes it difficult to put in place monitoring procedures, labor inspections provide an avenue for determining whether MCWs are facing labor rights violations [114]. Additionally, it is important that any inspections that take place are separate from immigration procedures, to not put workers at risk of deportation, and that they follow the ILO Labor Inspection Convention (No. 81) [111]. For Israel, social workers already provide visits to homes; however, these should occur more frequently, and should also focus not just on the quality of care provided but also on the working conditions of MCWs [39]. This is especially important for MCWs who assist people in need of support that have a high degree of dependency [48].

Relating to the theme *valuing care work*, both the Italian and Israeli governments should run education, information and awareness-raising campaigns around the value of paid and unpaid care work and the work provided by migrant workers. This will assist in combatting harmful stereotypes and assumptions present in the care and migration spheres and would likely help to reduce the negative assumptions around class disparities presented in the theme *perceptions on class asymmetries*. Likewise, societies which have more gender-responsive public policies have been shown to hold more gender-equal values and to support increased equality in the division of paid and unpaid work. Consequently, both governments should take a gender mainstreaming approach to the implementation of public policies [77].

It is important that in both Italy and Israel, MCWs, people in need of support and their relatives understand which rights MCWs are entitled to. Providing language training to MCWs will increase the likelihood that MCWs will understand their rights [71]. This will also help MCWs to be able to communicate with people in need of support and their family members, which should lead to higher-quality care being provided by MCWs. Likewise, it will also assist in reducing the power asymmetries between people in need of support and MCWs who were identified in the themes *being part of the family* and *perceptions on class asymmetries* [39].

### 4.4. Areas for Future Research

Almost all of the articles focused on Italy included in this review were qualitative. While this is useful in gaining an in-depth understanding of the interactions between MCWs and other actors, more quantitative research is needed to gain a better understanding of the magnitude of labor rights issues experienced by MCWs.

Few of the studies in Italy samples included people in need of support or their family members. The theme *being part of the family* shows that MCWs are often heavily reliant on people in need of support and their family members. Consequently, it is important that future research includes analysis on the experiences of people in need of support and their family members with MCWs. This will assist in increasing the sustainability of MCW markets, by ensuring that the challenges faced by each member of the MCW–person in need of support–informal caregiver triad can be overcome. A large number of articles focused on Israel included either MCW–person in need of support dyads or MCW–person in need of support–informal caregiver triads, with most of these dyads and triads having a quantitative focus. Consequently, more qualitative research on these dyads and triads would assist in gaining a better understanding of these relationships in this country context and beyond.

Looking at the mapping of articles against key phrases in the MFTCM, for Israel few articles included an analysis of the gender dimension at the macro level. As gender norms are deeply ingrained in the field of care work, future research is needed to shed light on how these care norms shape the interactions between MCWs and other actors in MCW markets. In addition, few study samples included men MCWs, and none explored the experiences of people that identify as non-binary or genderqueer. Further research is therefore needed to gain a greater understanding on whether these groups experience any specific challenges in the care sector.

### 4.5. Limitations

Only one researcher conducted the data extraction and analysis for this review. While the researcher used a structured approach to conduct this review, there is a chance that some data may have been missed or excluded. Moreover, because the researcher used reflexive thematic analysis, the development of themes was shaped by the work and educational backgrounds of the researcher [74]. Consequently, if this review was conducted again by other researchers, the developed themes may be different. Likewise, this review only included articles written in English, due to language limitations of the researcher. There may be relevant peer-reviewed articles in other languages that could provide further evidence on the topic of this review.

This article only analyses migrant care work models from the macro and micro levels; therefore, the role of some actors at the meso level were not included and properly considered in this review. Almost all of the articles included only focused on the role of the country of destination in developing care worker markets. Because care markets are transnational, it is also important to consider the role of countries of origin in the development of a more comprehensive understanding of this specific sector.

All of the articles reviewed in this article were published in academic journals. There is still the need for a more comprehensive review of grey literature that relates to migrant care work in these two countries. Due to the difficulties that migrant workers face in accessing decent work opportunities, there are a large number of organizations that provide support services to migrant workers. Since these organizations often produce reports and research for programming, a comprehensive review of existing research could help to increase the knowledge base on strategies and activities that can be helpful to improve care outcomes for migrant workers, people in need of support, and informal caregivers.

Despite these limitations, this work contributes, also thanks to its comparative approach, to enhance our understanding of the main areas in which research efforts should focus upon to find ways to improve the conditions of MCWs at a cross-national level.

## 5. Conclusions

This study provides a review of the most recent contributions to the fields of labor migration and health concerning the MCW markets in Italy and Israel. While there have been many studies in each country that detail the labor rights violations experienced by MCWs, this is the first review that develops themes around the underlying causes of these violations. By thematically analyzing the findings of recent studies and current gaps in existing knowledge, this scoping review assists in building the groundwork for the development and implementation of policy, strategies, practice and research to improve the rights and migration experiences of MCWs.

In the wider context, the findings of this scoping review are not only applicable to Italy and Israel, but may also apply to other MCW worker markets, especially in countries with familial care regimes. The methodology used in this scoping review could be used as a template to conduct further cross-national analyses on MCW markets in other countries. This would assist in building a comparable understanding of the common challenges and best practices around maintaining decent work opportunities for MCWs worldwide.

One commonality between Italy and Israel, and one of the largest challenges to overcome in both countries, is that care work—whether it be paid or unpaid—is routinely undervalued. At the macro level, this undervaluation of care work has contributed to a lack of political will to develop long-term sustainable solutions to create or monitor decent work standards for MCWs. Instead, both countries have used MCWs as a low-cost solution to fill long-term care gaps. At the micro level, this undervaluation has led to power imbalances between MCWs and people in need of care and their family members. This often results in MCWs being expected to work hours beyond those contractually allowed, having little to no time off, and experiencing emotional, physical, and sexual abuse.

Consequently, while policy measures are an important part of creating decent work opportunities for MCWs, these measures in themselves do not lead to increased rights for workers if these rights are not being enforced or people are not aware of them. The MCW markets in Italy and Israel do not need to be synonymous with exploitation and abuse. To provide better living and working conditions for MCWs, it is important that the Italian and Israeli governments work together alongside organizations that work on care and migration issues, local government officials, people in need of care and their family members, MCWs themselves, and researchers in order to develop new strategies and measures to enforce and maintain the rights of MCWs and to increase the recognition of the value of care work.

## Figures and Tables

**Table 1:** Summary of articles included in this review.

Study	Design	Field of Journal	Country Focus	Sample	Study Aim
Ayalon et al., 2015 [63]	Qualitative	Health Services Research and Policy	Israel	17 CRs ^1^, 16 family members ^2^, 20 MCWs ^3^ and 20 nurses	Understand the role of live-in MCWs in providing social care to patients in hospitals
Fusco et al., 2015 [49]	Quantitative	Geriatrics and Gerontology	Italy	506 CRs	Determine the impact of how being assisted by MCWs affects rehospitalization rates
Green and Ayalon, 2015 [64]	Quantitative	Gerontology	Israel	338 MCWs, 224 CRs, and 442 family members	Examine the extent to which people in need of support, family members, and MCWs are familiar with the rights of live-in MCWs
Mazuz, 2015 [65]	Qualitative	Global Health	Israel	1 triad (CR, family member, and MCW)	To analyze the use of somatic care practices by live-in MCWs
Meyer, 2015 [50]	Qualitative	Anthropology	Italy	MCWs—does not specify how many	Examine how MCWs negotiate their work lives
Ayalon and Roziner, 2016 [66]	Quantitative	Ageing Mental Health	Israel	23 triads (MCWs, CRs and family members)	Evaluate the satisfaction of relationships between people in need of support, their family members, and home care workers
Barbabella et al., 2016 [29]	Quantitative	Gerontology	Italy	438 CRs–primary family caregiver dyads	Investigate the socio-economic predictors of hiring MCWs
Boccagni, 2016 [51]	Qualitative	Social Politics	Italy	30 MCWs	To explore women MCWs mediation between different forms of well-being and to understand how these dimensions are understood, experienced and/or displaced while abroad
Green and Ayalon, 2016 [39]	Quantitative	Interpersonal Violence	Israel	187 MCWs	Explore help-seeking behaviours among MCWs who have experienced work-related abuse
Kemp and Kfir, 2016 [23]	Qualitative	Social Problems	Israel	15 NGO ^4^ staff members	Explain how civil society actors have mediated between the bio-political contradiction that MCWs are wanted workers but as unwanted mothers
Baldassar, Ferrero and Portis, 2017 [53]	Qualitative	Global Studies in Culture and Power	Italy	8 MCWs and 10 CRs and family members	Explain how kinning processes between MCWs and people in need of support develops
Cordini and Ranci, 2017 [52]	Qualitative	Social Policy	Italy	Content analysis of the public discourse in newspapers	Provide evidence on how market dynamics have allowed governments to shift the responsibility of providing home care to MCWs
Palumbo, 2017 [37]	Qualitative	Immigrant and Refugee studies	Italy	3 MCWs, 4 judicial and law enforcement authorities, 3 lawyers, 4 policymakers, 4 government representatives, 5 representatives of NGOs, 3 social workers, 2 trade unionists and 2 experts	Analysis of why exploitation in the domestic work sector is rarely acknowledged or addressed with polices around trafficking and exploitation
Rugolotto, Laroto, and van der Geest, 2017 [38]	Qualitative	Migration, Health and Social Care	Italy	20 MCWs, and 5 family members	Describe how migration affects the care being provided to people in need of support
Scrinzi, 2017 [54]	Qualitative	Western European Politics	Italy	20 party members of a political party	Examine the relationship between anti-immigration politics and the racialized and gendered division of care work
Boccagni, 2018 [55]	Qualitative	Housing Studies	Italy	165 MCWs	Analyze how live-in MCWs feel in their everyday lives abroad
Bonatti and Muniandy, 2018 [56]	Qualitative	Migration Studies	Italy and Malaysia	16 MCWs in Italy and 15 MCWs in Malaysia	Explain how migrant women develop and pursue their aspirations by examining the institutional limitations they face
Cherubini, Geymonat, and Marchetti, 2018 [57]	Qualitative	Participation and Conflict	Italy, Colombia, the Philippines, and Taiwan	Policy analysis of laws	Show how the ILO Domestic Workers Convention (No. 189) has been incorporated or resisted in local contexts
Green and Ayalon, 2018 [48]	Quantitative	Health Policy Research	Israel	338 MCWs and 185 Israeli care workers	Assess the working conditions and prevalence of abuse and exploitation faced by live-in MCWs and live-out local care workers
Nicolescu, 2018 [58]	Qualitative	East Central Europe	Italy	34 MCWs	Discuss how migrating to work in the care sectors is a transborder continuity of autonomy and employment practices that survive socialism
Bronstein, 2019 [67]	Qualitative	Documentation	Israel	20 MCWs	Examine the life stories of MCWs by analyzing different aspects of information behaviour that has emerged from their narratives through a transnational perspective
Brown, 2019 [45]	Qualitative	Feminist theory	Israel/Palestine	15 employers of MCWs ^5^	Examine the politics of the MCW–employer relationship as it unfolds within the Jewish-Israeli home
Cohen-Mansfield, 2019 [68]	Mixed methods—qualitative and qualitative	Gerontology	Israel	111 family members, 61 CRs, and 98 MCWs	Describe the social engagement care provided by live-in MCWs for frail older adults in comparison with the wishes of people in need of support and their families’ wishes for this care
Golan and Babis, 2019 [69]	Qualitative	Information, Communication and Society	Israel	800 Facebook posts	Explain how social networking site expressions shape an occupational community of temporary migrant workers
Ranci and Arlotti, 2019 [60]	Mixed methods—qualitative and qualitative	Policy and Society	Italy	60 key informants and INPS ^6^ data	Show how non-take up rates of health services can be explained by individually situated decisions taken by beneficiaries based on cost-benefit evaluations that are rooted in social attitudes shaped by existing institutional contexts
Nicolescu, 2019 [61]	Qualitative	Anthropology and Aging	Italy	34 MCWS and 24 employers of MCWs	Explore the success of the migrant in the family model and the mechanisms that bond MCWs and people in need of support in a mutual dependency
Solari, 2019 [59]	Qualitative	Sociology	Italy	61 MCWs and 39 adult children whose parent(s) were abroad	Uncover the meanings that MCWs and their non-migrant children assign to monetary and social remittances
Shinan-Altman and Ayalon, 2019 [70]	Quantitative	Ageing Mental Health	Israel	338 MCWs and 185 Israeli care workers	Examine the perceived control among live-in MCWs and live-out local care workers and identify the factors that contribute to this perceived control
Scrinzi, 2019 [44]	Qualitative	Immigrant and Refugee Studies	Italy	10 managers of the social cooperatives	Examine how strategies adopted by managers at social cooperatives challenge dominated gendered constructs of care work
Teshuva et al., 2019 [71]	Mixed methods—qualitative and qualitative	Ageing and Society	Israel	116 MCWs and 73 CRs	Explore the quality and the nature of relationships between live-in MCWs and people in need of support
Vianello, Finotelli and Brey, 2019 [62]	Qualitative	Ethnic and Migration Studies	Italy and Spain	10 MCWs in Italy and 10 MCWs in Spain	Investigate the process of residence permit renewals among migrants
Casanova, Tur-Sinai and Lamura, 2020 [34]	Qualitative	Ageing and Social Policy	Italy and Israel	Long-term care experts—12 in Israel, 27 in Italy	Identify the challenges and responses that have been adopted or should be adopted to improve long- term care provision in Italy and Israel
Holler, 2020 [72]	Qualitative	Social Policy and Administration	Israel	30 CRs	Examine the lived experience of people claiming disability benefits

^1^ People in need of support, ^2^ Family members of people in need of support, ^3^ Migrant care workers, ^4^ Non-Governmental Organization, ^5^ Includes people in need of support and/or their family members, ^6^ National Institute for Social Security.

**Table 2:** Mapping of key concepts in reviewed articles.

Key Concepts	Italy	Israel
Macro Level		
Gender Regime	[37,52,53,54,55,59,62]	[45,48,65]
Migration Regime	[37,38,52,54,56,61,62]	[23,34,39,64,67,69]
Care Regime	[29,34,37,38,44,49,50,51,52,53,54,55,58,60,61,62]	[34,39,45,48,65,68,69,72]
Employment Regime	[37,50,57,58,61,62]	[39,48,64,65,69]
Micro Level		
Gender	[37,44,50,51,53,55,59]	[23,39,45,48,65,68]
Power/Class Asymmetry	[37,44,50,51,55,59,61]	[23,45,48,63,64,65,66,67,68,69,70,71]
Culture, Ethnicity and Religion	[37,38,44,50,55,56]	[39,45,48,64,65,67,68,71]

## Data Availability

No new data were created or analyzed in this study. Data sharing is not applicable to this article.

## Appendix A

**Table A1:** Preferred Reporting Items for Systematic Reviews and Meta-Analyses Extension for Scoping Reviews (PRISMA-ScR) Checklist.

Section	Item	Prisma-Scr Checklist Item	Reported On Page #
**Title**			
Title	1	Identify the report as a scoping review.	1
**Abstract**			
Structured summary	2	Provide a structured summary that includes (as applicable): background, objectives, eligibility criteria, sources of evidence, charting methods, results, and conclusions that relate to the review questions and objectives.	1
**Introduction**			
Rationale	3	Describe the rationale for the review in the context of what is already known. Explain why the review questions/objectives lend themselves to a scoping review approach.	4–5
Objectives	4	Provide an explicit statement of the questions and objectives being addressed with reference to their key elements (e.g., population or participants, concepts, and context) or other relevant key elements used to conceptualize the review questions and/or objectives.	5
**Methods**			
Protocol and registration	5	Indicate whether a review protocol exists; state if and where it can be accessed (e.g., a Web address); and if available, provide registration information, including the registration number.	5
Eligibility criteria	6	Specify characteristics of the sources of evidence used as eligibility criteria (e.g., years considered, language, and publication status), and provide a rationale.	6
Information sources *	7	Describe all information sources in the search (e.g., databases with dates of coverage and contact with authors to identify additional sources), as well as the date the most recent search was executed.	6
Search	8	Present the full electronic search strategy for at least 1 database, including any limits used, such that it could be repeated.	28
Selection of sources of evidence †	9	State the process for selecting sources of evidence (i.e., screening and eligibility) included in the scoping review.	6
Data charting process ‡	10	Describe the methods of charting data from the included sources of evidence (e.g., calibrated forms or forms that have been tested by the team before their use, and whether data charting was done independently or in duplicate) and any processes for obtaining and confirming data from investigators.	7
Data items	11	List and define all variables for which data were sought and any assumptions and simplifications made.	7, 11
Critical appraisal of individual sources of evidence §	12	If done, provide a rationale for conducting a critical appraisal of included sources of evidence; describe the methods used and how this information was used in any data synthesis (if appropriate).	N/A
Synthesis of results	13	Describe the methods of handling and summarizing the data that were charted.	14
**Results**			
Selection of sources of evidence	14	Give numbers of sources of evidence screened, assessed for eligibility, and included in the review, with reasons for exclusions at each stage, ideally using a flow diagram.	6–7
Characteristics of sources of evidence	15	For each source of evidence, present characteristics for which data were charted and provide the citations.	7–14
Critical appraisal within sources of evidence	16	If done, present data on critical appraisal of included sources of evidence (see item 12).	N/A
Results of individual sources of evidence	17	For each included source of evidence, present the relevant data that were charted that relate to the review questions and objectives.	7–14
Synthesis of results	18	Summarize and/or present the charting results as they relate to the review questions and objectives.	15–21
**Discussion**			
Summary of evidence	19	Summarize the main results (including an overview of concepts, themes, and types of evidence available), link to the review questions and objectives, and consider the relevance to key groups.	21–24
Limitations	20	Discuss the limitations of the scoping review process.	25
Conclusions	21	Provide a general interpretation of the results with respect to the review questions and objectives, as well as potential implications and/or next steps.	25–26
**Funding**			
Funding	22	Describe sources of funding for the included sources of evidence, as well as sources of funding for the scoping review. Describe the role of the funders of the scoping review.	26

JBI = Joanna Briggs Institute; PRISMA-ScR = Preferred Reporting Items for Systematic reviews and Meta-Analyses extension for Scoping Reviews. * Where sources of evidence (see second footnote) are compiled from, such as bibliographic databases, social media platforms, and Web sites. † A more inclusive/heterogeneous term used to account for the different types of evidence or data sources (e.g., quantitative and/or qualitative research, expert opinion, and policy documents) that may be eligible in a scoping review as opposed to only studies. This is not to be confused with information sources (see first footnote). ‡ The frameworks by Arksey and O’Malley (6) and Levac and colleagues (7) and the JBI guidance (4, 5) refer to the process of data extraction in a scoping review as data charting. § The process of systematically examining research evidence to assess its validity, results, and relevance before using it to inform a decision. This term is used for items 12 and 19 instead of “risk of bias” (which is more applicable to systematic reviews of interventions) to include and acknowledge the various sources of evidence that may be used in a scoping review (e.g., quantitative and/or qualitative research, expert opinion, and policy document). From: Tricco AC, Lillie E, Zarin W, O’Brien KK, Colquhoun H, Levac D, et al. PRISMA Extension for Scoping Reviews (PRISMAScR): Checklist and Explanation. Ann Intern Med. 2018, 169, 467–473. doi:10.7326/M18-0850.

## Appendix B. Boolean Operators Used

“gender regime” OR “employment regime” OR “migration regime” OR “care regime” OR “welfare state” OR “welfare regime” OR “care culture” or “gender” OR “class” OR “ethnicity” OR “culture” OR “migration” OR “nationality” OR “rights” OR “immigration” AND (“Italy” OR “Israel”) AND “migrant” AND (“care worker” OR “domestic worker” OR “careworker” OR “carer” OR “caregiver”).

## Appendix C. References Including for Mapping and Thematic Analysis

1. Ayalon, L.; Halevy-Levin, S.; Ben-Yizhak, Z.; Friedman, G. Personal home care workers’ role in hospital: a qualitative study. *Journal of Health Services Research and Policy* **2015**, *20*, 217–223, doi:10.1177/1355819615577807.
2. Fusco, S.; Corsonello, A.; Chiatti, C.; Fabbietti, P.; Salerno, G.; De Bonis, E.; Corica, F.; Lattanzio, F. Migrant care workers and rehospitalization among older patients discharged from acute care hospitals. *Geriatr. Gerontol. Int.* **2015**, *15*, 196–203, doi:10.1111/ggi.12254.
3. Green, O.; Ayalon, L. Whose right is it anyway? Familiarity with workers’ rights among older adults, family caregivers, and migrant live-in home care workers: implications for policy and practice. *Educational Gerontology* **2015**, *41*, 471–481.
4. Mazuz, K. Elderly care between global and local services: The use of somatic care practices. *BMJ Case Rep.* **2015**, *2015*, doi:10.1136/bcr-2015-211055.
5. Meyer, P. Relations of Care: The Contexts for Immigrant Care Workers in Northern Italy. *Antropol. Work Rev.* **2015**, *36*, 2–12, doi:10.1111/awr.12054.
6. Ayalon, L.; Roziner, I. Satisfaction with the relationship from the perspectives of family caregivers, older adults and their home care workers. *Aging Ment. Health* **2016**, *20*, 56–64, doi:10.1080/13607863.2015.1020412.
7. Barbabella, F.; Chiatti, C.; Rimland, J.M.; Melchiorre, M.G.; Lamura, G.; Lattanzio, F. Socioeconomic Predictors of the Employment of Migrant Care Workers by Italian Families Assisting Older Alzheimer’s Disease Patients: Evidence From the Up-Tech Study. *J. Gerontol. B Psychol. Sci. Soc. Sci.* **2016**, *71*, 514–25, doi:10.1093/geronb/gbv045.
8. Boccagni, P. Searching for well-being in care work migration: Constructions, practices and displacements among immigrant women in Italy. *Soc. Polit.* **2016**, *23*, 284–306, doi:10.1093/sp/jxv031.
9. Green, O.; Ayalon, L. Whom Do Migrant Home Care Workers Contact in the Case of Work-Related Abuse? An Exploratory Study of Help-Seeking Behaviors. *J. Interpers. Violence* **2016**, *31*, 3236–3256, doi:10.1177/0886260515584347.
10. Kemp, A.; Kfir, N. Wanted Workers but Unwanted Mothers: Mobilizing Moral Claims on Migrant Care Workers’ Families in Israel. *Social Problems* **2016**, *63*, 373–394, doi:10.1093/socpro/spw016.
11. Baldassar, L.; Ferrero, L.; Portis, L. ‘More like a daughter than an employee’: the kinning process between migrant care workers, elderly care receivers and their extended families. *Identities* **2017**, *24*, 524–541, doi:10.1080/1070289X.2017.1345544.
12. Cordini, M.; Ranci, C. Legitimising the care market: The social recognition of migrant care workers in Italy. *Journal of Social Policy* **2017**, *46*, 91–108, doi:10.1017/S0047279416000398.
13. Palumbo, L. Exploiting for Care: Trafficking and Abuse in Domestic Work in Italy. *J. Immigr. Refugee Stud.* **2017**, *15*, 171–186, doi:10.1080/15562948.2017.1305473.
14. Rugolotto, S.; Larotonda, A.; van der Geest, S. How migrants keep Italian families Italian: badanti and the private care of older people. *Int. J. Migr. Health Soc. Care* **2017**, *13*, 185–197, doi:10.1108/ijmhsc-08-2015-0027.
15. Scrinzi, F. Caring for the elderly in the family or in the nation? Gender, women and migrant care labor in the Lega Nord. *West Eur. Polit.* **2017**, *40*, 869–886, doi:10.1080/01402382.2017.1287447.
16. Boccagni, P. At home in home care? Contents and boundaries of the ‘domestic’ among immigrant live-in workers in Italy. *Hous. Stud.* **2018**, *33*, 813–831, doi:10.1080/02673037.2017.1367366.
17. Bonatti, V.; Muniandy, P. Defiant aspirations: Migrant women’s struggles for stability and upward mobility in Naples and Kuala Lumpur. *Migr. Stud.* **2018**, *8*, 113–130, doi:10.1093/migration/mny039.
18. Cherubini, D.; Geymonat, G.G.; Marchetti, S. Global rights and local struggles: The case of the ILO convention N.189 on domestic work. *Partecip. Confl.* **2018**, *11*, 717–742, doi:10.1285/i20356609v11i3p717.
19. Green, O.; Ayalon, L. Violations of workers’ rights and exposure to work-related abuse of live-in migrant and live-out local home care workers—a preliminary study: implications for health policy and practice. *Isr J Health Policy Res* **2018**, *7*, 32, doi:10.1186/s13584-018-0224-1.
20. Nicolescu, G. From border fetishism to tactical socialism. *East Cent. Eur.* **2018**, *45*, 279–299, doi:10.1163/18763308-04502005.
21. Bronstein, J. A transitional approach to the study of the information behavior of domestic migrant workers: A narrative inquiry. *J. Doc.* **2019**, *75*, 314–333, doi:10.1108/JD-07-2018-0112.
22. Brown, R.H. Reproducing the national family: kinship claims, development discourse and migrant caregivers in Palestine/Israel. *Fem. Theory* **2019**, *20*, 247–268, doi:10.1177/1464700119833039.
23. Cohen-Mansfield, J.; Golander, H.; Iecovich, E.; Jensen, B. Social Engagement Care for Frail Older Persons: Desire for It and Provision by Live-In Migrant Caregivers. *J. Gerontol. B Psychol. Sci. Soc. Sci.* **2019**, *74*, 1062–1071, doi:10.1093/geronb/gbx052.
24. Golan, O.; Babis, D. Towards professionalism through social networks: constructing an occupational community via Facebook usage by temporary migrant workers from the Philippines. *Inf. Commun. Soc.* **2019**, *22*, 1230–1252, doi:10.1080/1369118X.2017.1411520.
25. Ranci, C.; Arlotti, M. Resistance to change. The problem of high non-take up in implementing policy innovations in the Italian long-term care system. *Policy Soc.* **2019**, *38*, 572–588, doi:10.1080/14494035.2019.1619995.
26. Nicolescu, G. Keeping the elderly alive global: Entanglements and embodied practices in long-term care in southeast Italy. *Anthropology Aging* **2019**, *40*, 77–93, doi:10.5195/aa.2019.202.
27. Solari, C.D. Transnational moral economies: The value of monetary and social remittances in transnational families. *Curr. Sociol.* **2019**, *67*, 760–777, doi:10.1177/0011392118807531.
28. Shinan-Altman, S.; Ayalon, L. Perceived control among migrant live-in and local live-out home care workers in Israel. *Aging Ment. Health* **2019**, *23*, 189–195, doi:10.1080/13607863.2017.1401584.
29. Scrinzi, F. Beyond “Women’s Work”: Gender, Ethnicity, and the Management of Paid Care Work in Nonprofit Domiciliary Services in Italy. *J. Immigr. Refugee Stud.* **2019**, *17*, 441–456, doi:10.1080/15562948.2018.1538472.
30. Teshuva, K.; Cohen-Mansfield, J.; Iecovich, E.; Golander, H. Like one of the family? Understanding relationships between migrant live-in care workers and older care recipients in Israel. *Ageing and Society* **2019**, *39*, 1387–1408, doi:10.1017/S0144686 × 1800003X.
31. Vianello, F.A.; Finotelli, C.; Brey, E. A slow ride towards permanent residency: legal transitions and the working trajectories of Ukrainian migrants in Italy and Spain. *J. Ethn. Migr. Stud.* **2019**, doi:10.1080/1369183X.2019.1590187.
32. Casanova, G.; Principi, A.; Lamura, G. Social innovation in long-term care: Lessons from the Italian case. *Int. J. Environ. Res. Public Health* **2020**, *17*, doi:10.3390/ijerph17072367.
33. Holler, R. Material, stigmatic, and agentic dimensions in the experience of claiming disability benefits: The Israeli case. *Soc. Policy Adm.* **2020**, doi:10.1111/spol.12578.
